# A Novel Technique for Substrate Toughening in Wood Single Lap Joints Using a Zero-Thickness Bio-Adhesive

**DOI:** 10.3390/ma17020448

**Published:** 2024-01-17

**Authors:** Shahin Jalali, Catarina da Silva Pereira Borges, Ricardo João Camilo Carbas, Eduardo André de Sousa Marques, Alireza Akhavan-Safar, Ana Sofia Oliveira Ferreira Barbosa, João Carlos Moura Bordado, Lucas Filipe Martins da Silva

**Affiliations:** 1 Institute of Science and Innovation in Mechanical and Industrial Engineering (INEGI), Rua Dr. Roberto Frias, 4200-465 Porto, Portugal; 2 Departamento de Engenharia Mecânica, Faculdade de Engenharia, Universidade do Porto, Rua Dr. Roberto Frias, 4200-465 Porto, Portugallucas@fe.up.pt (L.F.M.d.S.); 3 Centro de Recursos Naturais E Ambiennte (CERENA), Instituto Superior Técnico, University of Lisbon, 1049-001 Lisbon, Portugal

**Keywords:** wooden joints, toughened joint, transverse strength, delamination, polyurethan bio-adhesives

## Abstract

In contemporary engineering practices, the utilization of sustainable materials and eco-friendly techniques has gained significant importance. Wooden joints, particularly those created with polyurethan-based bio-adhesives, have garnered significant attention owing to their intrinsic environmental advantages and desirable mechanical properties. In comparison to conventional joining methods, adhesive joints offer distinct benefits such as an enhanced load distribution, reduced stress concentration, and improved aesthetic appeal. In this study, reference and toughened single-lap joint samples were investigated experimentally and numerically under quasi-static loading conditions. The proposed research methodology involves the infusion of a bio-adhesive into the wooden substrate, reinforcing the matrix of its surfaces. This innovative approach was developed to explore a synergetic effect of both wood and bio-adhesive. The experimentally validated results showcase a significant enhancement in joint strength, demonstrating an 85% increase when compared to joints with regular pine substrates. Moreover, the increased delamination thickness observed in toughened joints was found to increase the energy absorption of the joint.

## 1. Introduction

The recent concerted drive towards reducing the impact of fossil-based products has spurred the development and increasingly larger adoption of bio-based materials [1,2,3]. This endeavor seeks to usher in a more sustainable era, where the utilization of tree and plant-derived products assumes a pivotal role. This is particularly true in the creation of eco-friendly load-bearing structures, enabled by the fabrication of composites comprising natural fibers, such as flax, jute, and palm tree. Within this context, the significance of wood as a naturally sourced structural material comes to the fore. Across the annals of history, wood has been explored for diverse daily applications due to its status as a replenishable natural material [4,5,6]. Noteworthy attributes such as its versatility in assuming various shapes, coupled with its impressive durability, resistance to wear and environmental factors, mechanical robustness, relatively low weight, global abundance, and economic viability, position it as a valid and sustainable material choice. Moreover, wood’s adaptability to distinct environmental conditions, including temperature fluctuations, loading rates, and moisture influences, has led to its widespread use in structural applications, especially in the civil engineering field [7,8,9]. Wood thus stands out as a natural composite material but, and akin to most composites, design of complex wooden structures is not trivial, since it exhibits sensitivity to the introduction of holes and notches. Traditional joining methods like riveting and bolting are unsuitable for wood due to its composition [10]. In light of this, adhesive bonding emerges as a suitable technique for joining wood parts. This method circumvents the creation of stress concentrations and establishes a broader, more uniform bonded area. Adhesive joints are primarily designed to operate under shear forces, enhancing the material’s resistance. However, in the most commonly used joint configuration, the single-lap joint (SLJ), the overlapped and offset substrates can bend to ensure an alignment of the load path. This alignment generates a flexural moment at the ends of the overlap, leading to the formation of stress concentrations at the overlap ends. Consequently, the stress distribution within the adhesive becomes a combination of both shear and tensile forces. This combination of loading modes highlights the complex interplay that adhesive joints must withstand when subjected to varying loads and conditions [11,12]. As a result, the failure load and the failure mode of the joint become contingent on not only the cohesive properties of the adhesive but also that of its substrates. In adhesive joints involving wood substrates, the contribution of peel loading is particularly concerning, since it leads to delamination between distinct grain layers and ultimately causes joint failure. To counteract this issue, multiple corrective approaches have been proposed, such as wood densification, substrate toughening, and even physical modifications of the adhesive [3]. These processes aim to tackle the challenge of delamination and bolster the integrity of the adhesive and the wood substrate.

Multiple recent research works have been devoted to improving the efficacy of wooden adhesive joints, integrating bio-adhesives and wood-based substrates in joint configurations. The failure mode and load capacity of joints are significantly influenced by the properties of both the adhesive and the substrate. As discussed above, peel stress is a critical factor that affects the strength and failure of composite materials, especially when their matrix is weak. This is because the fibers within the matrix may not reach their peak load before the matrix fails [13,14]. Therefore, any method that enhances the peel strength of wood directly contributes to the overall strength of the joints [15]. In contrast, the strength of a given bio-adhesive and its additives is still an unexplored path, since these materials are still mostly used in non-structural applications.

Baumberger et al. [16] studied films with up to 30% kraft lignin and starch, created using extrusion and thermal molding, seeking to improve the performance of these natural materials. The mechanical properties of the composite were evaluated through tensile tests at two humidity levels. The study found that lignin increased the strength and strain-to-failure of the composite at 58% relative humidity (RH) for lignin contents up to 20%. It was observed that lignin reduced the affinity of the films to water, and this behavior was attributed to a combination of the hydrophilic starch matrix and hydrophobic lignin. This aspect is of concern in adhesives with additives since the adhesive matrix is generally more hydrophilic than the reinforcement, making the adhesive/reinforcement interface a favorable pathway for water diffusion [17].

One of the methods that can improve the strength of SLJ is to use a matrix with high toughness in the transverse direction, which can enhance the resistance to peel stress. This method can be combined with other methods that aim to reduce the local stress concentration in the composite adherends [18].

Stucki et al. [19] investigated the use of bio-based bonding additives, especially kraft lignin and its derivatives, to enhance the moisture resistance and bond strength of friction-welded wood joints. Results of this work show that kraft lignin has the best performance among the tested biomolecules, and that its molecular structure and thermal behavior affect the bonding quality. The paper also discusses the possible bonding mechanisms involved in the friction welding process, and suggests further research directions to optimize the lignin-based bonding additives.

Shang et al. [14] presented a methodology aimed at mitigating delamination issues in adhesive joints featuring composite substrates. The investigation encompassed both experimental and numerical aspects, evaluating parameters such as joint strength, failure modes, and stress distribution within the SLJs. The toughened composite material yielded a substantial 22% increase in joint strength and, remarkably, a shift in failure mode from adherend delamination to cohesive failure within the adhesive. The findings of this study suggest that the toughened composite material holds the potential to effectively mitigate delamination in adhesive joints involving composite substrates while enhancing overall performance.

Conventional wood joints have several drawbacks, such as low transverse strength, poor durability, and high environmental impact of synthetic adhesives. Therefore, alternative methods are needed to improve the performance and sustainability of wood joints. to overcome these challenges a novel and effective method was proposed to enhance the strength and durability of wood joints by increasing the peel and transverse strength of the substrate using a polyurethane-based adhesive derived from natural resources. This method could improve the mechanical properties and resistance to environmental factors of wood joints, as well as reduce the carbon footprint and toxicity of the adhesive. By applying the bio-adhesive on the surface of the wood substrates, the shear and peel strength of the SLJs can increase, as well as the absorbed energy during failure. A series of experiments were conducted to compare the performance of the proposed method with that of conventional methods. The peel and transverse strength of the wood and testing SLJ joints under static loading conditions were measured. The proposed method significantly increased the peel and transverse strength of the wood joints, as well as their durability and stability under various environmental factors. The results suggest that the method can provide a viable solution to the challenges and limitations of conventional wood joints. The study contributes to the advancement of wood engineering and the development of eco-friendly adhesives. A numerical model was also developed to clarify the mechanisms behind the mechanical behaviors that were observed.

## 2. Material

### 2.1. Bio-Adhesive

A polyurethane-based bio-adhesive, derived from 70% renewable biomass sources, such as vegetable oils, according to the ASTM D6866 standard [20], was used, which exhibits excellent affinity for wood and holds significant potential as a viable alternative to synthetic adhesives. This is a novel monocomponent system with a 100% solids content, which means it does not contain any solvents or low molecular weight oligomers that could compromise its performance. It has an average molecular weight of 5200 and three terminal aliphatic isocyanate groups (-NCO) that react with moisture and active hydrogen atoms on the surfaces of the substrates, forming strong covalent bonds. The adhesive layer thickness is a key parameter for the lap joint performance, and it was optimized for our experimental setup. The adhesive also has a unique composition that replaces the conventional propylene oxide (PO) based polyhydroxyl alcanoates with ethylene oxide (EO) based ones in the “3 legs” of the structure. This results in aliphatic polar bio-derived structures that enhance the wetting and physical adhesion to polar substrates. The nominal viscosity of the adhesive at 25 °C is 8 Poise, which facilitates its application and curing.

**Figure 1:**
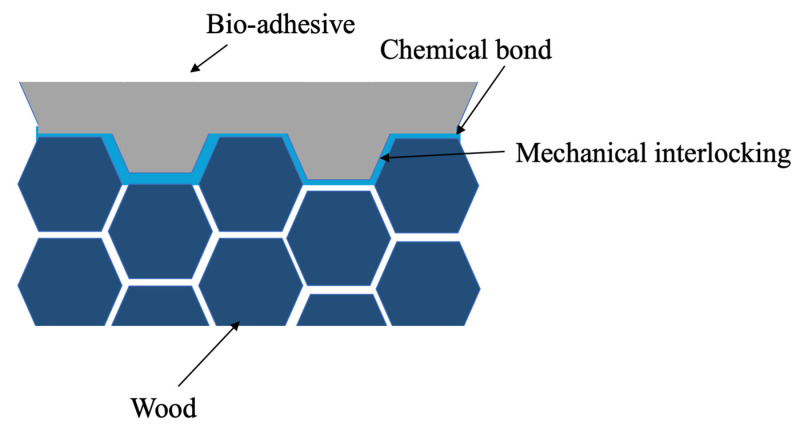
Schematic molecular bonds of moisture curing adhesive.

The bio-adhesive was developed by the team of Professor João Bordado at Instituto Superior Técnico. The bio-adhesive is not yet commercially available, but it shows consistent performance with typical moisture-curing polyurethane adhesives. Furthermore, due to the zero-thickness curing process of the adhesive, it lends itself well to be used in many wooden applications. The bio-adhesive is produced in an irreversible reaction, without humidity, in a reactor under a nitrogen atmosphere, and heating is achieved with a thermal oil coil. The bio-adhesive uses an aliphatic isocyanate as a basis, which contains 70% plant matter, as these are more easily biodegradable. Manufacturing the bio-adhesive is estimated to consume 15–20% less energy than those derived from petroleum. The bio-adhesive consists of pentamethylene diisocyanate and polyisocyanate, which form strong chemical bonds with the wood substrate by reacting with its hydroxyl (OH) groups. The curing process of the bio-adhesive is accelerated by increasing the humidity of the substrates. To achieve uniform curing of the bio-adhesive in the joints, all samples were maintained at consistent moisture levels. The bio-adhesive offers multiple benefits due to the synergistic interplay between its components and the wood’s OH groups, as well as the influence of humidity. The bio-adhesive not only enhances the mechanical interlocking and the physical and mechanical bonds within the joints, which are typical for normal adhesives, but also provides superior chemical bonding effects. The combination of these factors contributes to the overall strength of the joints (see Figure 1).

This bio-adhesive requires a curing process at 100 °C for a duration of 8 h, followed by a post-curing period of 48 h at room temperature. This consistent curing procedure was applied to all tested samples, to ensure comparability. The mechanical properties of the adhesive were previously characterized under quasi-static conditions (1 mm/min) by Jalali et al. [21], and can be found in Table 1.

### 2.2. Substrate

The main substrate under investigation consisted of pine timber obtained from Portuguese sources. Initially, the timber was sourced in beams with a length of 1000 mm, which was subsequently cut into smaller pieces with dimensions suitable for the joint and bulk sample configurations. The thickness of the wood was standardized at 6 mm, while the width was set at 25 mm.

The selection of pine wood as the primary material was based on various factors, including its widespread availability, cost-effectiveness, and desirable mechanical properties, such as strength, stiffness, and durability. It is important to note that wood, being a complex and heterogeneous material, exhibits properties that can vary due to factors such as species, growth conditions, grain slope and size, defects, knots, shakes, and age. The substrates, selected from the beams that had the most symmetrical grains and no noticeable damage, had a moisture content ranging between 12% and 18%, which was guaranteed by the supplier. The moisture content of the wood was within the standard range of 8–25% by weight for different types and uses of wood. The wood was considered dry, as it had a moisture content of 19% or less, which is the maximum value for sawn lumber design. The moisture content of the wood was an important factor for its properties and performance, as it affected its dimensional stability, strength, durability, and biological resistance.

**Figure 2:**
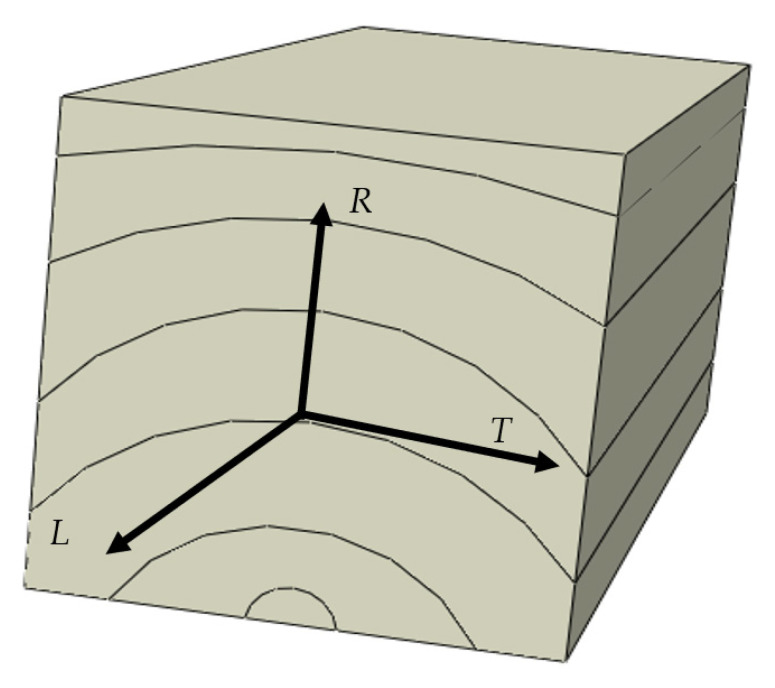
Wood beam directions.

To further emphasize the significance of the chosen substrate, Oliveira et al. [22] conducted extensive testing on pine wood and compiled the results, the elastic properties Young’s modulus (*E*), Poisson’s ratio (*ν*), and shear modulus (*G*) are summarized in Table 2. All the wood directions are shown in Figure 2.

### 2.3. Toughened Substrate

The substrate edges were toughened by infusing the bio-adhesive in the pine wood substrates. This method was chosen due to the higher strength exhibited by the bio-adhesive in comparison to the wood matrix (lignin). 

The adhesive penetration process in the wood involved two main steps. In the first step, all the surfaces of the substrate were thoroughly wetted with the bio-adhesive using a brush. This ensured that the adhesive was evenly distributed and absorbed by the entire surface area of the wood. The purpose of this step was to facilitate effective bonding between the adhesive and the wood.

**Figure 3:**
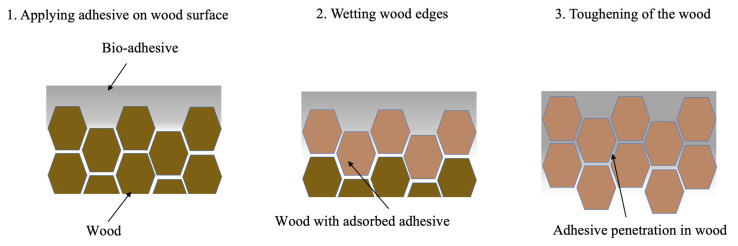
Toughening procedures.

Following the initial wetting step, the substrate was then soaked in the adhesive pot for 1 h. This extended soaking allowed for optimal penetration and interaction between the bio-adhesive and the wood substrate, further strengthening the bond. Figure 3 presents a schematic diagram, illustrating the step-by-step procedure of wood toughening through compression.

Also, the density of the toughened samples was measured after the curing process of the bio-adhesive. The results indicated that the average density of the reference wood was 0.56 ± 0.03 g/cm^3^ and that the bio-adhesive increased the wood density by 4%.

## 3. Experimental Procedure

### 3.1. Bulk Testing

The mechanical behavior of the reference, non-toughened, pine wood and toughened pine wood was assessed by conducting traction tests on 1 mm thick pine wood samples in both the fiber direction and matrix direction. The reference pine wood samples underwent a simple sanding process to ensure smooth surfaces and minimize the presence of microcracks and surface stress concentrations.

**Figure 4:**
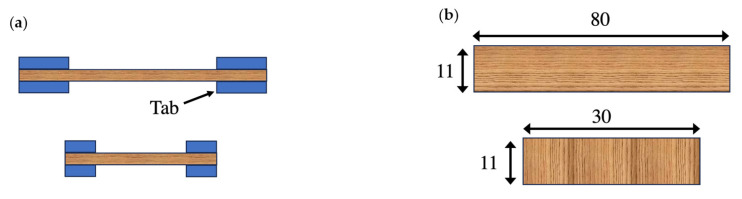
Pine wood bulk sample, tested sample (**a**), geometry and dimension of the tested wood (**b**). (Dimensions are in mm.).

The toughened samples were prepared by applying the previously mentioned toughening procedures on a 1 mm thick wood plate. Wooden end tabs were then bonded to the ends of the samples, as depicted in Figure 4a. The geometry and dimensions of the bulk samples can be observed in Figure 4b. Through this testing approach, a comprehensive comparative analysis was conducted between the toughened and reference samples, thus providing valuable insights into the mechanical performance of the toughened layer.

### 3.2. Joint Testing

**Figure 5:**
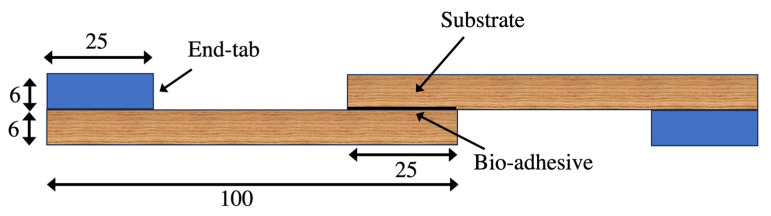
SLJ geometry and dimensions. (Dimensions are in mm.)

SLJ samples were prepared with an overlap length of 25 mm, ensuring direct bonding of the substrates to meet the zero-thickness requirement of the bio-adhesive. To achieve uniform pressure distribution along bonding area, clamps were used to apply a constant level of pressure to the joints. In order to protect the substrates from damage caused by the applied pressure, 2 mm thick aluminum plates were employed, as illustrated in Figure 5. To facilitate the easy debonding of the joint from the aluminum plates and clamps, a mold-release agent was applied the aluminum surfaces.

Figure 5 shows the joint geometries and dimensions of the SLJs. It is worth noting that, assuming no edge effects, the stress and strain conditions along the width direction should remain consistent for SLJs with different widths. As a result, the failure load of the SLJs is expected to be directly proportional to the width. For the purpose of maintaining consistency and facilitating comparison, a sample width of 25 mm was selected in this study. Previous research has already investigated the impact of overlap length, with several authors conducting tests and comparisons using different values [23,24].

#### Surface Preparation

The successful adhesion of the pine wood substrate relies on a correct preparation of the bonding surfaces [4]. To achieve the desired zero-thickness bond with the adhesive, the surfaces underwent a sanding process, employing 400-grade sandpaper. This step ensured that the surfaces were smooth and uniform, providing an optimal foundation for bonding.

Following the sanding procedure, compressed air was employed to thoroughly clean the wood surfaces, effectively removing any loose particles that could impede proper adhesion. The presence of dust particles on the surface creates a barrier, hindering the adhesive’s ability to form a robust bond and potentially resulting in weak or non-bonded joints.

To further ensure the absence of contaminants on the wood surfaces, an additional cleaning step was carried out, using a solvent (acetone) to remove impurities such as oils waxes and other organic substances from the surface. Once the wood surfaces were properly prepared, a layer of adhesive was applied to the bonding area of each substrate before they were assembled. A total of ten SLJs were manufactured simultaneously. The entire setup was then transferred to an oven, where it was subjected to an appropriate cycle to cure the adhesive.

After the curing process, excess adhesive was removed and fixing tabs at the ends of the substrates were bonded using Araldite AV138 (Duxford, UK), a two-component epoxy adhesive that cures at room temperature for 24 h.

### 3.3. Testing Condition

In real-world applications, adhesive joints experience various types of loads, including quasi-static conditions. For the quasi-static testing phase, an INSTRON 3367(Norwood, MA, USA) universal testing machine equipped with a 30 kN load cell was utilized at ambient room temperature. A constant cross head displacement rate of 1 mm/min was maintained throughout the testing process. For each specific joint the configuration was tested, and a load-displacement curve was generated by the machine. To ensure the statistical validity of the results, four repetitions were performed for each configuration under analysis. At least three samples were tested for each configuration.

## 4. Experimental Results and Discussion

### 4.1. Bulk Testing

**Figure 6:**
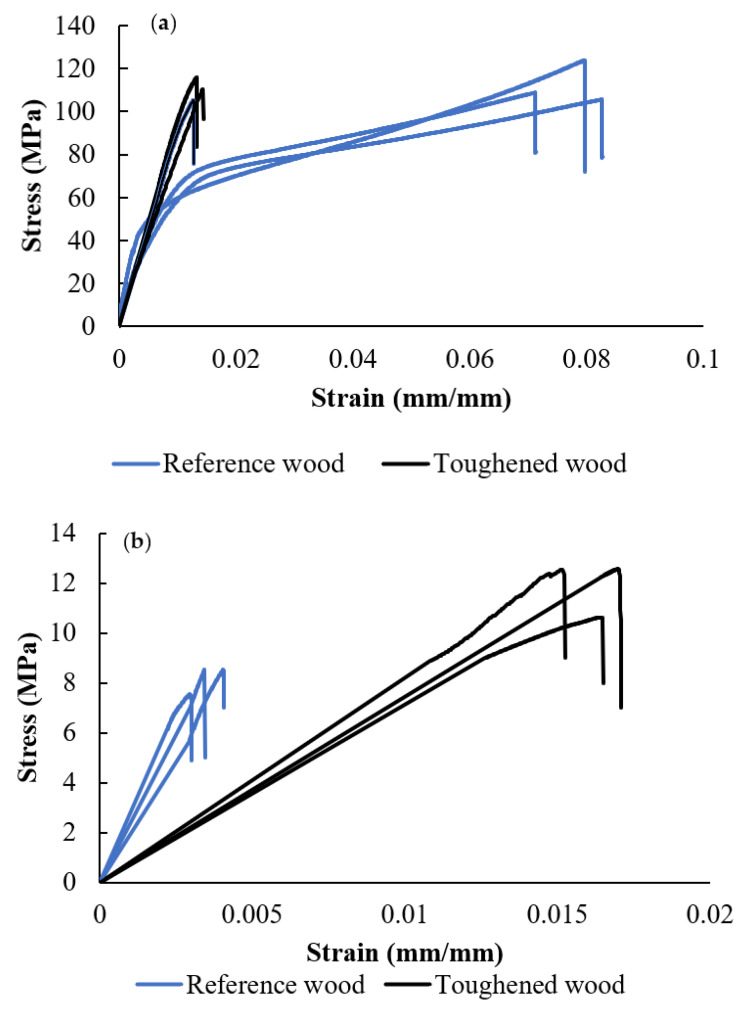
Typical stress-strain behavior of bulk samples. Fiber direction (**a**), matrix direction (**b**).

The effect of toughening on the mechanical properties of pine wood was investigated along both the fiber and matrix directions. The results revealed that adhesive penetration increased the strength of the toughened wood by 10% when tested along the fiber direction. However, adhesive penetration did not have a significant effect on the elastic modulus of the wood along the fiber direction, as shown in Figure 6a. On the other hand, the strain at failure decreased by 60% in these samples, and the adhesive made the wood significantly more brittle along this direction. In contrast, adhesive penetration notably enhanced the wood strength along the matrix direction. The superior strength of the adhesive, compared to the wood matrix, resulted in penetration between the wood cells and the formation of chemical bonds. This led to a remarkable 180% increase in both the failure strength and the failure strain of the wood along the matrix direction. Moreover, the failure strain and the absorbed energy of the toughened wood increased by two and seven times, respectively, compared to the reference wood samples (as shown in Figure 6b). The testing results of the toughened ply showed significant improvements in its strength compared to the reference wood. The elastic properties of the samples are presented in Table 3.

### 4.2. Joint Testing

**Figure 7:**
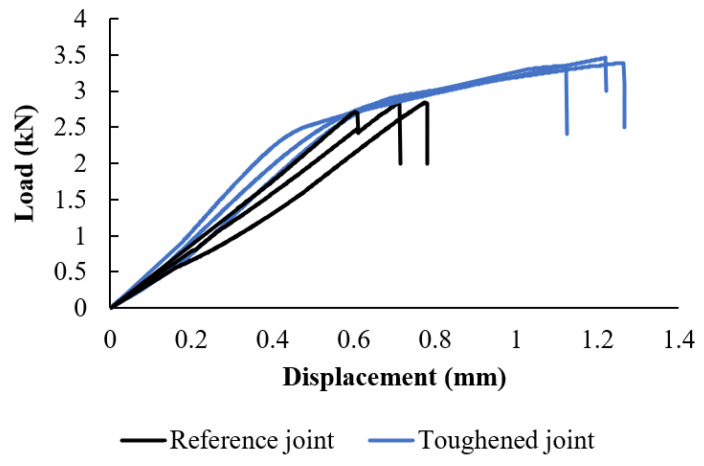
Typical load-displacement of SLJ.

The experimental results depicted in Figure 7 display the load-displacement behavior of the joints under quasi-static conditions. It is observed that the toughened joints exhibited a higher failure load, with an increase of approximately 20%, compared to the reference joints. Furthermore, the stiffening effect on the joints was notable, as indicated by an increase in stiffness of approximately 38%. Of particular interest is the behavior of the toughened joints at the point of failure. When the toughened joints reached the failure load of the reference joints, the toughening process induced plastic deformation within the joint structure. As a result, the displacement at failure increased by approximately 85%, indicating a greater capacity for energy absorption before failure. This increase in absorbed energy was substantial, amounting to approximately 170% compared to the reference joints. The improved energy absorption capability of the toughened joints highlights their enhanced resilience and ability to dissipate energy effectively.

The nonlinear behavior of the joints results in a significantly larger area underneath the curve leading to substantial enhancement in the quantity of energy absorbed during testing. Additionally, it is noteworthy that the process of toughening has a more pronounced effect on the absorbed energy of the joint than on its failure load, as the amount of energy absorbed has increased by around 230%.

#### 4.2.1. Fracture Surfaces

**Figure 8:**
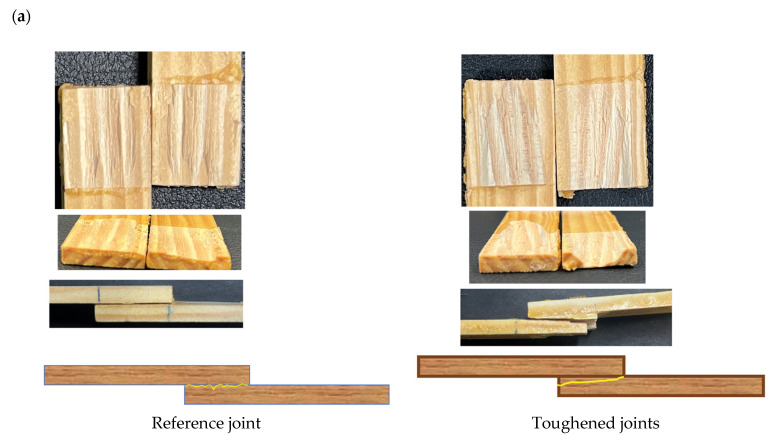

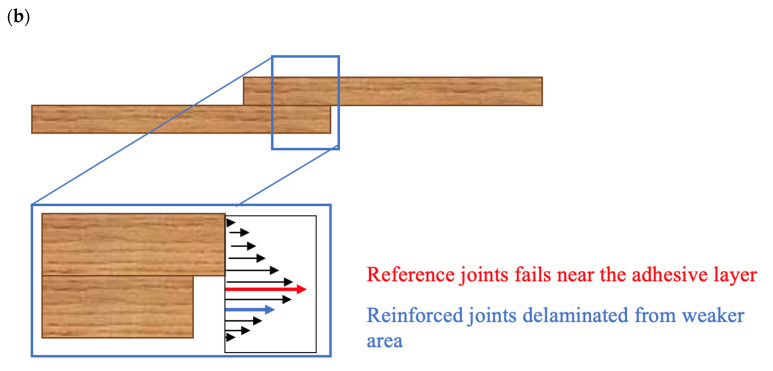
Fracture surface of SLJ (**a**), fracture behavior explanation (**b**).

Figure 8a provides digital images of the fracture surfaces that were analyzed to gain insight into the behavior of the joints. The examination of these images revealed that delamination of the wood was the primary failure mode observed in both the toughened and reference joints. This finding indicates that the adhesive achieved good adhesion and could be cured effectively.

However, a notable difference was observed in the toughened joints, indicating a significant change from the behavior seen in the reference joints. In the reference joints, delamination typically occurred at a relatively shallow depth within the wood substrate. This meant that the failure was concentrated in the surface layers of the wood. In contrast, the toughened joints revealed a notably different failure pattern. Within the toughened joints, an intriguing phenomenon unfolded. The failure predominantly transpired within the non-toughened region of the wood substrate. It is worth emphasizing the significance of this observation as while the non-toughened core of the wood bore the brunt of the failure, the outer plies, which had experienced enhanced adhesion due to adhesive penetration, were found to be more resilient. This particular behavior was instrumental in driving up the failure load of the toughened joints. The key to this phenomenon lies in the stress distribution along the overlap. At the ends of the overlap, where stresses tend to concentrate, stress levels approached the failure threshold of the wood. This stress concentration effect was instrumental in the change in failure mode observed.

Figure 8a presents the difference fracture behavior of the joints. In the toughened joint, the crack path assumed a more complex and notably deeper trajectory. This complexity led to a substantial increase in the amount of energy required for the joint to reach its failure point. On the flip side, in the reference joint, the delamination path remained relatively uniform, with the crack predominantly propagating horizontally along the overlap. However, in the toughened joint, the crack’s propagation followed a more intricate path. Not only did it traverse the overlap horizontally, but it also exhibited a tendency to change direction vertically, interacting with different grain orientations, and even propagating vertically through the thickness of the wood substrate.

Figure 8b provides a schematic explanation of this failure mechanism. It visually demonstrates how the toughening process leads to a redistribution of the failure zone, with the failure occurring in the non-toughened area while the toughened outer plies retained their integrity. This behavior highlights the positive effect of the toughening technique, which enhanced the load-bearing capacity and resistance to failure of the joints.

#### 4.2.2. Scanning Electron Microscopy (SEM) Analysis

**Figure 9:**
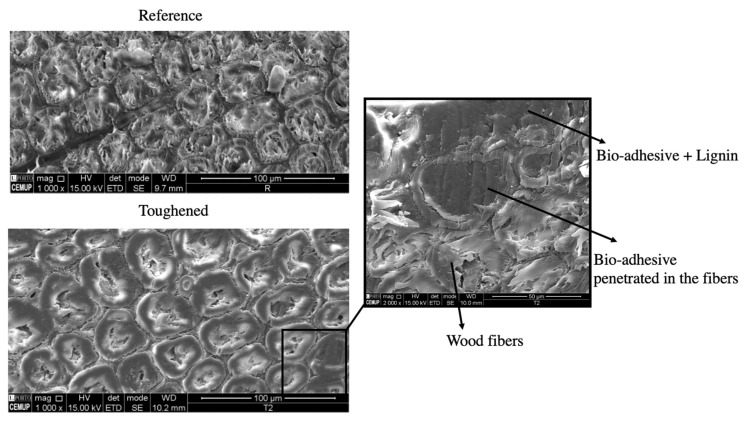
Scanning electron microscopy analysis.

To gain further insight into the behavior of the joints, SEM analysis was conducted to examine the adhesive’s penetration and its interaction with the wood substrates. SEM analyses were performed using a JEOL JSM 6301F/Oxford INCA Energy 350/Gatan Alto 2500 microscope (Tokyo, Japan) at CEMUP (University of Porto, Portugal). The microscope was operated at an accelerating voltage of 20 kV and a working distance of 10 mm. A secondary electron detector was used to image the adhesive composition and the wood structure. Since the analyzed samples were not conductive, they were coated with a thin film of gold (Au)/palladium (Pd) alloy, by sputtering, using the SPI module sputter coater equipment. The sputtering time was 120 s and the current was 15 mA. As depicted in Figure 9, reference substrate and edges of toughened substrate were compared. The SEM images revealed the effective penetration of the adhesive into the wood. This analysis confirmed that not only the wood matrix was reinforced, but also that the adhesive penetrated the wood fibers. The SEM analysis provided visual evidence of the adhesive’s ability to penetrate and interact with the wood fibers, enhancing the overall strength and toughness of the joint. By saturating the outer layers of the wood fibers, the adhesive created a reinforced zone that contributed to the joint’s improved performance. This reinforcement allowed the wood fibers to undergo plastic deformations, enhancing the joint’s ability to withstand applied loads and reducing the likelihood of premature failure.

## 5. Numerical Analysis

### 5.1. Numerical Details

To validate the experimental results and to support the investigation into the failure mechanism, a static finite element analysis was conducted using ABAQUS v2017 software. In order to simplify the analysis and reduce computational time, a two-dimensional (2D) model was employed, assuming the stress distribution along the width of the SLJ to be uniform.

The primary objective of this analysis was to predict the load-displacement behavior and failure modes of the joints. The numerical results were evaluated by considering a representative experimental curve that could capture the average response of the samples.

The wood samples in all tested cases exhibited elastic deformation, and thus, linear elastic behavior was assumed for the substrates that the model was meshed using quadrilateral elements with four nodes and plane stress conditions. Also, to predict the initiation and propagation of potential damages and delamination of the substrates, a cohesive zone model (CZM) was employed. Four-node cohesive quadrilateral elements were used to model the behavior of the substrates, employing cohesive elements with a triangular (bilinear) traction-separation law based on the mechanical and cohesive properties of pine wood Table 2 and Table 4. The CZM elements were introduced to both the reference and toughened substrates, by considering the delamination thickness observed in the experiments, in order to effectively simulate the delamination phenomenon.

**Figure 10:**
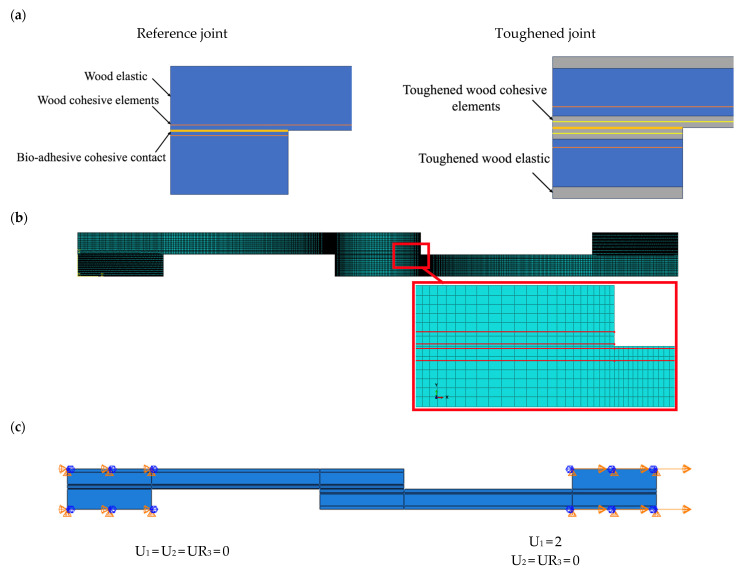
(**a**) Partition, (**b**) mesh used in SLJ simulation, (**c**) boundary condition.

Cohesive elements were placed at a distance of 0.1 mm from the substrate interface for the reference joints, and for toughened joints the cohesive elements were placed in thicknesses of 0.1 and 1.3 mm from the interface of the substrates in order to predict any possible delamination in the toughened wood or core wood. The thickness of cohesive elements was measured from the average thickness of delamination obtained in the experiments. The adhesive behavior was simulated by using cohesive contact between two substrates assuming the bio-adhesive properties. In regions with significant stress gradients, the element sizes were refined by reducing the mesh size. The mesh was distributed along the substrates and bondline in the x-direction using single and double biasing, where element sizes of 0.01 mm and 0.05 mm were employed. Along the end tabs (see Figure 10a,b) a uniform mesh distribution was applied. The assumed boundary condition is illustrated in Figure 10c where the left side of SLJ was fixed and the right side subjected to a displacement of 2 mm.

### 5.2. Numerical Results

**Figure 11:**
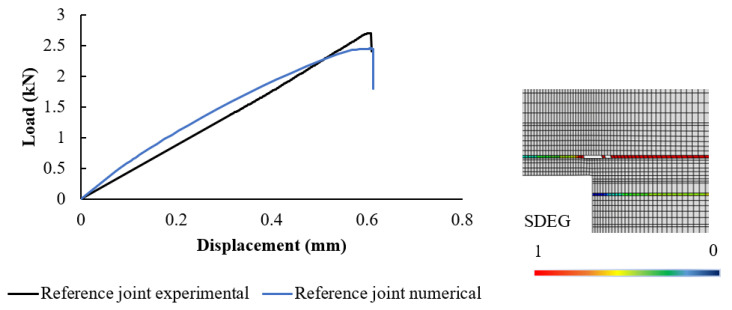
Numerical analysis of reference joint. The image on the right shows the damage distribution, where the SDEG is the damage index, which varies between 0 (no damage) and 1 (complete failure).

In Figure 11, a comparison between the numerical and experimental load-displacement data for the reference joint is presented. The predicted failure load from the numerical model closely matched the results of the experiments. This congruence serves as a validation of the practicality and accuracy of the mechanical properties attributed to the materials within the simulation. Just as in the experiments, the cohesive zones situated beneath the initial wood plies displayed a notable and extensive level of degradation. In stark contrast, the adhesive layer exhibited minimal signs of degradation. The cracks started where the first layer of wood meets the second layer along the overlap’s edge. This spot is weaker because of stress considerations at the overlap ends. So, this is where failure often begins in these joints. This localized vulnerability played a pivotal role in the initiation of the failure process, shedding light on a critical aspect of the joint’s mechanical behavior.

**Figure 12:**
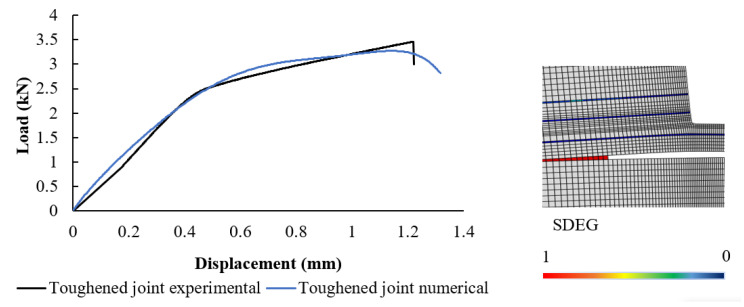
Toughened joint numerical analysis. The image on the right shows the damage distribution, where the SDEG is the damage index, which varies between 0 (no damage) and 1 (complete failure).

As illustrated in Figure 12, the numerically derived failure mechanism and load-displacement curve for toughened joints display a good agreement with the experimental results at the point of failure load. This close correlation between the numerical models and the experimental data highlights the reliability of the simulation. The numerical simulation has proven its efficacy in accurately predicting the experimental failure mechanism, as can be observed in Figure 8. Notably, the observed failure mechanism in toughened joints involves the cohesive failure of the wood’s cohesive layer.

Both experimental and numerical findings lead to the conclusion that the reinforcement of the substrate through the application of bio-adhesive leads to a noticeable augmentation in the failure load. Furthermore, a significant increase in delamination thickness is observed in cases where the substrate has been toughened. This dual enhancement process, which results in an elevated failure load and larger delamination thickness, shows the effectiveness and future potential of the process of reinforcing the substrate with bio-adhesive.

## 6. Conclusions

This study has successfully introduced a novel and practical approach to enhance the loading capacity of joints with wood substrates and bonded with bio-adhesives. The reinforcement strategy involves infusing bio-adhesive into the wooden substrate, reinforcing the matrix near the surface. The method for producing the toughened substrate demonstrated consistent and repeatable results, establishing its feasibility for real-world applications.

The experimental assessment of SLJs utilizing the toughened substrate yielded promising outcomes. Notably, the substrate’s delamination thickness experienced a significant increase, showing an 85% rise in joint strength compared to conventional SLJ samples with regular pine substrates.

The exploration of failure behavior, augmented failure load, and improved absorbed energy was supported by the development of a finite element method model. This modeling approach effectively minimized the stress concentration at the overlap edges and efficiently distributed stresses over a wider area. As a result, the joints exhibited a significant increase in failure load.

This comprehensive study not only presents a practical technique for enhancing wooden joint performance but also offers insights into the underlying mechanics of the observed improvements. The combination of bio-adhesive infusion, experimental validation, and advanced modeling techniques marks a significant stride toward enhancing the robustness and reliability of wooden joints in structural applications.

The main findings of this study were:

▪ The toughened substrates increased the delamination thickness and the joint strength by 85% compared to regular pine substrates.
▪ The failure behavior, failure load, and absorbed energy of the joints were explored and supported by a finite element method model.
▪ The model reduced the stress concentration at the overlap edges and increased the stress distribution over a wider area.
▪ The study presented a practical technique for enhancing wooden joint performance using bio-adhesive infusion.
▪ The study offered insights into the underlying mechanics of the observed improvements.

## Figures and Tables

**Table 1:** Bio-adhesive properties [21].

Young’s Modulus (MPa)	Tensile Strength (MPa)	Mode I Fracture Energy (N/mm)	Mode II Fracture Energy (N/mm)
197.09 ± 9.76	3.27 ± 0.14	0.33 ± 0.03	1.27 ± 0.1

**Table 2:** Nine elastic properties of the pine wood along longitudinal (*L*), radial (*R*) and tangential (*T*) directions [22].

*E_L_* (GPa)	*E_R_* (GPa)	*E_T_* (GPa)	*ν_LT_*	*ν_LR_*	*ν_TR_*	*G_LR_* (GPa)	*G_LT_* (GPa)	*G_TR_* (GPa)
12.0	1.9	1.0	0.5	0.4	0.3	1.1	1.0	0.3

**Table 3:** Elastic behavior of bulk samples.

		Young’s Modulus (MPa)	Tensile Strength (MPa)	Strain at Failure (%)
Fiber direction	Reference	12.3 ± 1.2	93.2 ± 4.2	7.6 ± 0.3
	Toughened	11.9 ± 1.8	102.3 ± 8.2	1.3 ± 0.1
Matrix direction	Reference	2.1 ± 0.1	8.1 ± 0.3	0.4 ± 0.1
	Toughened	0.8 ± 0.1	11.8 ± 0.8	1.6 ± 0.1

**Table 4:** Strength (σ) properties of pine wood [22].

$\sigma_{L}$ (MPa)	$\sigma_{R}$ (MPa)	$\sigma_{T}$ (MPa)	$\sigma_{LR}$ (MPa)	$\sigma_{LT}$ (MPa)	$\sigma_{RT}$ (MPa)
97.5	7.9	4.2	16.0	16.0	4.5

## Data Availability

Data are contained within the article.
